# Movement performance and movement difficulties in typical school-aged children

**DOI:** 10.1371/journal.pone.0249401

**Published:** 2021-04-08

**Authors:** Rujira Jaikaew, Nuntanee Satiansukpong

**Affiliations:** 1 Master Student, Faculty of Associated Medical Sciences, Department of Occupational Therapy, Chiang Mai University, Chiang Mai, Thailand; 2 Associate Professor, Faculty of Associated Medical Sciences, Department of Occupational Therapy, Chiang Mai University, Chiang Mai, Thailand; Jiangsu Normal University, CHINA

## Abstract

**Introduction:**

Developmental Coordination Disorder (DCD) is an impairment of executive motor skills. Children aged 7–10 years gradually develop effective movement that enables smooth performance in various daily self-care, academic and sport activities. The purpose of this study was to examine whether the Movement Assessment Battery for Children-Second Edition-Age Band 2, (MABC2-AB2), which is a western standardized test, could be used in Thai children for differentiating between movement performance and movement difficulties.

**Method:**

Three hundred and sixty typical Thai children aged 7–10 years old were recruited from three primary schools in Chiang Mai district, Thailand. The participants were divided into four age groups and tested using the Movement Assessment Battery for Children-Second Edition-Age Band 2-Thai version (MABC2-AB2-T).

**Results:**

Manual Dexterity, Aiming and Catching, and Balance rose with age increment. Older participants had better movement performance than younger ones. The results showed that 91.11 percent of the participants had typical movement, while 3.61 and 5.28 percent of them had movement difficulty and movement at risk, respectively. In addition, three test items: Drawing Trail, Walking Heel to Toe Forward, and Hopping on Mats had a ceiling effect when used for Thai children.

**Conclusion:**

The MABC2-AB2-T could be used to assess movement performance and movement difficulties in Thai children. About 9 percent of typical Thai children aged 7–10 years old needed early intervention. Administration of the three test items may need to be revised.

## Introduction

Competence in movement performance ranges from gross motor coordination and balance to fine motor coordination, which is necessary for 7–10 year-old typical school-aged children when participating in daily self-care, and academic and sport activities [1,2]. However, some children in this age group execute various motor tasks with difficulty [2,3]. Their movement performance falls far below that of their peers. They experience difficulties in coordination, maintaining posture and controlling body movements, which are critical in performing daily tasks in life [4,5]. These children are often diagnosed with Developmental Coordination Disorder (DCD) [6]. Many terms are used interchangeably for DCD such as clumsy child syndrome, childhood dyspraxia and specific developmental disorder of motor function [6].

Poor movement performance often reduces participation, e.g. in playing with peers at school, and joining sports clubs or home activities [7]. Consequently, less practice and less experience in various motor skills impact on self-esteem and self-confidence, which may create further problems, such as depression, anxiety and social isolation [8]. Thus, early detection of movement difficulties in children aged 7–10 years is essential in providing appropriate intervention.

The onset of DCD symptoms occurs during childhood [6]. Children with DCD are characterized by delayed development in gross and fine motor skill, which is not explained by intellectual developmental disorder, visual impairment or neurological conditions that affect movement [6]. These movement difficulties manifest commonly as clumsiness, e.g., dropping or bumping into objects, slow movement, and uncoordinated and imprecise movement skills in, for instance, handwriting, catching a ball, using scissors and playing sport [6].

The prevalence of DCD in children aged 5–11 years is 5–6 percent [6]. The reported prevalence of movement difficulty in Singapore, Columbia and Taiwan was 1.8, 3 and 3.5 percent, respectively, which was lower than that reported in the UK (5–6%), Spain (9.9%) and Brazil (17.8%) [9–13]. These differences might be due to the measurement tool used, cut-off point, culture and the children’s environment. Males are affected more than females with a ratio of between 2:1 and 7:1 [6].

Previous international studies often used the Developmental Coordination Disorder Questionnaire (DCDQ) [14], Bruininks-Oseretsky Test of Motor Proficiency, Second Edition (BOT-2) [15], and Movement Assessment Battery for Children-Second Edition (MABC-2) [1,16], as instruments for studying DCD. The DCDQ is a questionnaire that gathers information from teachers and parents, and not a direct measure of the child’s movement performance. The BOT-2 and MABC-2 are used often to measure the child’s movement performance, with the latter administered worldwide for detecting movement difficulty in 20–40 minutes, which is quicker than the time taken by the BOT-2 (40–60 minute).

Diagnosis of DCD is difficult in Thailand because it requires a standardized test, such as motor skill assessment using the MABC-2 [6], which is not translated into Thai or standardized. No previous studies have used the Movement Assessment Battery for Children-Second Edition-Age Band 2 (MABC2-AB2) to study movement performance and movement difficulties in Thai children. Therefore, this study aimed at using the MABC2-AB2 for this purpose. This study was carried out after a preceding one, from which the Thai version of the MABC2-AB2, and study of its cross-cultural validity and interrater reliability was obtained [17].

The MABC2-AB2 measures three movement domains: Manual Dexterity, Aiming and Catching, and Balance [16]. Manual Dexterity is the ability to use both dominant and non-dominant hands in a skillful and coordinated way in order to grasp and manipulate objects, write, and demonstrate small, precise movements. Manual Dexterity and Aiming and Catching skill increases with age and experience [1,18,19]. Aiming and Catching requires body part coordination, visual tracking and visuomotor control [20]. The Thai Physical Education curriculum for 10-year-olds contains learning two sports, which probably provides the opportunity for Thai children at this age to practice aiming and catching skills [21].

Balance depends on ability in the visual, somatosensory and vestibular system [22,23]. Research shows that Balance improves with age [24], and developmental improvements are noted between 2- and 12-year-olds [25]. A previous study of 165 healthy Brazilian children, aged 8–12 years old, showed that Balance develops and increases linearly with age [24]. Condon and Cremin studied Balance in 534 Irish typical children by using the One-Foot Balance test [26]. Their results showed that 7-year-olds were able to stand on one foot for between 8 and 32 seconds, while 10-year-olds could stand that way for 48 to 120 seconds, which indicated the trend of development. Dynamic control mechanism of Balance was different in children with DCD from that in typical children [27].

Previous studies, which used the MABC-2 in UK, Korea and Taiwanese children reported that boys had more movement difficulties than girls [9,11,28]. The gender ratio of boys to girls for UK and Korean Children was 1.9:1 [9] and 1.7:1 [28], respectively. In addition, the study of DCD in Korea reported that Korean children struggled prominently in Aiming and Catching skills [28]. A study in Japan reported that US American children aged between 7 and 10 years outperformed Japanese children in the same age group in Manual Dexterity, including threading tasks and making trails, but the opposite result was found in Balance. The ceiling effect of Balance was reported in Japanese children using the MABC2-AB2. A previous study, which tested 35 British children aged between 8 and 10 years old, found a ceiling effect in the Drawing Trail test [29]. The MABC2-AB2 has been used worldwide, but not in Thai children. Therefore, use of the MABC2-AB2 was the next step in carrying out a normative study of Thai children.

This study used the MABC2-AB2-T to investigate movement performance and movement difficulties in typically developed Thai school-aged children. The objectives were to 1) examine movement performance differences between 7- and 10-year-olds in Manual Dexterity, Aiming and Catching, and Balance, and 2) study whether the MABC2-AB2-T could be used with Thai Children.

## Materials and methods

This was a cross-sectional study with a protocol approved by the Ethics Committee, Faculty of Associated Medical Sciences, Chiang Mai University, Thailand (AMSEC-60EX-042). The participants and their parents signed an assent and consent form before participating in this study.

### Participants

Three-hundred and sixty typically developed Thai children were recruited from three schools in Muang district, Chiang Mai, Thailand, by using a stratified random and simple random sampling method. The inclusion criteria were (1) aged 7 years 0 month to 10 years 11 months, (2) no diagnosed physical disability, and (3) ability to understand all test instructions. The exclusion criterion was (1) having visual impairment (2) having a neurological condition affecting movement, e.g. cerebral palsy, muscular dystrophy and degenerative disorder, and (3) inability to complete the test. The participants were divided into 4 age groups: 7-year-olds; 8-year-olds; 9-year-olds; and 10-year-olds. Each child was tested individually in Manual Dexterity, Aiming and Catching, and Balance.

### Instrument

The Movement Assessment Battery for Children version 2-Age Band 2-Thai version (MABC2-AB2-T) was used for examining movement performance of the child. Permission for translation of the MABC2-AB2 [16] into the Thai language was granted by the Pearson Corporation. The psychometric properties of the MABC2-AB2-T were studied and previously reported in another study [17]. The MABC2-Age Band 2 (AB2: children aged 7–10 years) was translated into Thai from the source version of the MABC2 by using the following steps: forward translation, backward translation, panel discussion, and test of the prefinal version of the Thai-MABC2-AB2. Five occupational therapists checked the content validity of the test. Twenty-nine children, aged 7–10 years, were examined by two testers in order to establish interrater reliability [17]. The content validity ranged from 0.73 to 0.95 and interrater reliability from 0.71 to 1.00 [17].

Movement performance was divided into three categories: movement difficulty, movement at risk, and typical movement development according to the percentile rank (PR) score. Children with a PR score above the 15th percentiles on the MABC2—AB2-T were identified as those with typical development. Movement difficulty (DCD) referred to a PR at or below the 5th percentile. Movement at risk referred to a PR between the 6th and 15th percentile [16].

### Procedures

Two occupational therapists conducted the MABC2-AB2-T for each participant in a quiet room at school. The test was administered individually by following administration of the MABC2-AB2 [16] as follows:

1. Manual Dexterity Test. Each participant was tested in performing three tasks: Placing Pegs, Threading Lace and Drawing Trail. For Placing Pegs, each hand was tested in one attempted practice that involved placing six pegs in a blue board. Two actual trials were then conducted with each hand. For the Threading Lace and Drawing Trail; one attempted practice and two actual trials for each task were given by threading a red lace through four holes on a yellow lacing board, and then drawing a trail without error. Time to complete Placing Pegs and Threading Lace was recorded. Number of errors was recorded for the Drawing Trail [16].
2. Aiming and Catching Test. Catching with Two Hands and Throwing a Beanbag onto a Mat were tested. Five practice attempts were given for throwing a ball at a wall from a marked distance and catching the rebound, and for throwing a beanbag onto a circular orange target. Each task was tested by conducting two trials, and then 10 attempts for each test. The number of successful catches and throws was recorded [16].
3. Balance (One-Board Balance, Walking Heel-to-Toe Forward, and Hopping on Mats). For One-Board Balance, one attempted practice on each leg was carried out for 15 seconds, and then if the child could not stand on either leg for 30 seconds in the first actual trial, a second one was carried out. The balance on a board time was recorded for up to 30 seconds. Next, 4.5 meters of tape were placed on the floor, and one attempted practice of walking five steps heel-to-toe forward on it was taken, before two actual trials were performed. The number of correct consecutive steps was recorded. Lastly, one attempted practice of hopping on each leg was given, and then two actual trials for each leg. The number of correct consecutive hops on each leg was recorded [16].

### Data analysis

The data were analysed using the SPSS program for Windows version 17. Descriptive statistics such as mean, standard deviation and percentage were calculated. Inferential statistics: 1. the One-way Analysis of Variance (ANOVA) were analysed in order to compare the movement performance between the four age groups, 2.the Chi-square test was analysed to compare the prevalence distributed between genders.

## Results

Movement performance was explored in the 360 typically developed Thai children. The outliner data were taken out, which left 335 participants (171 boys and 164 girls) for the mean and standard deviation analysis. The demographic data of the participants are shown in Table 1. Movement performance of each age group in the Manual Dexterity, Aiming and Catching, and Balance subtests are shown in Tables 2–4, respectively.

**Table 1:** Demographic data of the participants (n = 335).

Age group	Gender	N	Age (years-months)		
			Min.	Max.	$\bar{x}$±SD
**7-year-olds** (7–0–7–11)^a^	Boys	39	7–3	7–11	7–8 ± 0.19
	Girls	35	7–2	7–11	
**8-year-olds** (8–0–8–11)	Boys	38	8–0	8–11	8–7 **±** 0.23
	Girls	42	8–0	8–11	
**9-year-olds** (9–0–9–11)	Boys	47	9–0	9–11	9–7 **±** 0.25
	Girls	43	9–0	9–11	
**10-year-olds** (10–0–10–11)	Boys	47	10–0	10–11	10–7 **±** 0.22
	Girls	44	10–0	10–11	

a: 7 years 0 month to 7 years 11 months.

**Table 2:** Movement performance in the Manual Dexterity test of each age group (n = 335).

Age group	Manual Dexterity ($\bar{x}$±SD)			
	Placing Pegs (secs)		Threading Lace (secs)	Drawing Trail (no. errors)
	preferred hand	non-preferred hand		
**7-year-olds**	28.39 ± 4.10	32.36 ± 4.60	25.73 ± 5.02	0.28 ± 0.60
**8-year-olds**	26.69 ± 4.07	29.74 ± 4.27	23.46 ± 4.91	0.11 ± 0.39
**9-year-olds**	24.79 ± 3.66	27.94 ± 3.87	21.80 ± 4.42	0.07 ± 0.25
**10-year-olds**	24.51 ± 3.74	26.97 ± 3.87	20.29 ± 3.78	0.08 ± 0.30

**Table 3:** Movement performance in the Aiming and Catching subtest of each age group (n = 335).

Age group	Gender	n	Aiming and Catching ($\bar{x}$±SD)	
			Catching with Two Hands (no. catches)	Throwing Beanbag onto Mat (no. hits)
**7-year-olds**	Boy	39	5.97 ± 2.25	6.41 ± 1.58
	Girl	35	5.69 ± 2.16	6.09 ± 1.82
	Total	74	5.84 ± 2.20	6.26 ± 1.69
**8-year-olds**	Boy	38	5.89 ± 3.21	7.11 ± 1.75
	Girl	42	5.43 ± 3.18	7.36 ± 1.46
	Total	80	5.65 ± 3.18	7.24 ± 1.60
**9-year-olds**	Boy	47	6.53 ± 2.55	7.74 ± 1.62
	Girl	43	5.33 ± 2.83	6.93 ± 1.73
	Total	90	5.96 ± 2.74	7.36 ± 1.71
**10-year-olds**	Boy	47	8.32 ± 1.61	8.23 ± 1.25
	Girl	44	6.82 ± 2.14	7.48 ± 1.42
	Total	91	7.59 ± 2.02	7.87 ± 1.38

**Table 4:** Movement performance in the Balance subtest of each age group (n = 335).

Age group	Gender	n	Balance ($\bar{\bar{x}}$±SD)				
			One-board Balance (secs)		Walking Heel to Toe Forward (no. steps)	Hopping on Mats (no. jumps)	
			best leg	other leg		Best leg	Other leg
**7-year-olds**	Boy	39	19.59 ± 3.96	13.44 ± 10.04	13.56 ± 2.56	4.77 ± 0.53	4.46 ± 0.72
	Girl	35	27.29 ± 5.91	23.43 ± 8.70	14.31 ± 2.29	4.80 ± 0.40	4.60 ± 0.55
	Total	74	23.23 ± 9.10	18.16 ± 10.63	13.92 ± 2.45	4.78 ± 0.47	4.53 ± 0.64
**8-year-olds**	Boy	38	23.45 ± 7.93	14.63 ± 9.16	12.39 ± 3.59	4.92 ± 0.27	4.63 ± 0.63
	Girl	42	24.86 ± 6.26	15.60 ± 8.32	13.95 ± 2.46	4.95 ± 0.21	4.64 ± 0.61
	Total	80	24.19 ± 7.09	15.14 ± 8.69	13.21 ± 3.13	4.94 ± 0.24	4.64 ± 0.62
**9-year-olds**	Boy	47	25.74 ± 7.09	17.36 ± 9.70	13.66 ± 2.97	4.96 ± 0.20	4.77 ± 0.52
	Girl	43	27.67 ± 4.91	21.67 ± 8.95	14.67 ± 1.42	4.93 ± 0.33	4.72 ± 0.59
	Total	90	26.67 ± 6.19	19.42 ± 9.55	14.14 ± 2.40	4.94 ± 0.27	4.74 ± 0.55
**10-year-olds**	Boy	47	27.32 ± 5.56	20.17 ± 9.69	14.36 ± 2.06	4.98 ± 0.14	4.83 ± 0.43
	Girl	44	25.84 ± 6.79	20.61 ± 9.36	14.41 ± 1.78	4.91 ± 0.29	4.73 ± 0.45
	Total	91	26.60 ± 6.20	20.38 ± 9.48	14.38 ± 1.92	4.95 ± 0.22	4.78 ± 0.44

The comparison of Manual Dexterity using ANOVA showed a significant difference between the age groups (F = 6.046, *p* = 0.001) (Table 5). Further multiple comparison analysis showed a significant difference between 3 pairs (7- and 8-year-olds, 7- and 9-year-olds, and 7- and 10-year-olds). Older participants tended to use less time in Placing Pegs and Threading Lace, and make fewer errors in the Drawing Trail test, than younger ones (Table 2).

**Table 5:** Comparison of movement performance between the four groups using one-way ANOVA.

		Sum of Squares	Df	Mean Square	F	*p*.
**Manual Dexterity**	Between Groups	325.721	3	108.574	6.046	.001*
	Within Groups	5944.267	331	17.959		
	Total	6269.988	334			
**Aiming and Catching**	Between Groups	70.700	3	23.567	1.373	.251
	Within Groups	5682.333	331	17.167		
	Total	5753.033	334			
**Balance**	Between Groups	132.682	3	44.227	2.146	.094
	Within Groups	6820.942	331	20.607		
	Total	6953.624	334			

*significant difference at α = 0.05.

However, there were no significant differences between the age groups in Aiming and Catching (F = 1.373, *p* = 0.251) and Balance (F = 2.146, *p* = 0.094) (Table 5). The 10-year-olds obtained the best scores in the Aiming and Catching subtest, while the 7-, 8- and 9-year-olds showed a lower performance (Table 3). The 9- and 10-year-olds achieved similar and the highest score, respectively, in Balance. Two test items of Balance had a ceiling effect (Table 4).

Movement performance of the participants was separated into 3 categories: movement difficulty, movement at risk, and typical movement, according to their PR scores. The percentages of the participants in each category are shown in Table 6, and 91.11% of them had typical movement. Percentages of the other participants were for movement at risk (5.28%) and movement difficulty (3.61%), reaching almost 9 percent. Further analysis with the Chi-square test showed that the prevalence of movement difficulty, movement at risk, and typical movement was significantly different between boys and girls (χ^2^ = 0.11, p = 0.001).

**Table 6:** Percentages of male and female participants with movement difficulty, movement at risk, and typical movement (n = 360).

Subtests	% Difficulty			%At Risk			%Typical		
	Boy	Girl	B&G	Boy	Girl	B&G	Boy	Girl	B&G
Manual Dexterity	0	0	0	0.56	0	0.56	49.16	50.28	99.44
Aiming and Catching	1.67	1.38	3.05	1.11	0.56	1.67	46.94	48.33	95.27
Balance	0.27	0.56	0.83	1.94	1.11	3.05	47.50	48.61	96.11
Total	1.67	1.94	3.61	3.61	1.67	5.28	44.44	46.67	91.11

Note: Movement difficulty = score at and below the 5th percentile: movement at risk = score between the 6th and 15th percentile: typical movement = score above the 15th percentile.

## Discussion

The results of movement performance in Thai children aged 7–10 years old, in the areas of (1) Manual Dexterity, (2) Aiming and Catching, and (3) Balance, showed a trend of improvement with age increment. The data analysis using ANOVA showed a significant difference between the age groups only in Manual Dexterity, but not in the last two areas. Previous studies showed improvement of Manual Dexterity with age [18,19] as well as Aiming and Catching [1] and Balance [25,26]. Children aged 7–10 years old normally spend their time performing fine and gross motor activities. Cech and Martin mentioned that mastery of specific prehension skills was dependent on the amount of time spent practicing [30].

Movement is developed while a child interacts with his/her environment. A variety of movements are used, and the best solution of movement for each task is selected. Task demand and task complexity help the child to learn specific movement patterns. Experience and repetition refine certain movements to become more accurate and precise, resulting in fewer errors [27,31]. Most of the Thai children aged 10 years old obtained a full score in Aiming and Catching. The Thai Physical Education curriculum for this age contains learning two sports that probably provides more opportunities for practicing aiming and catching skills [21], in which Thai children have struggled prominently. The same problem was reported previously in Korean children [28]. Thai and Korean children might have a similar culture, and an environment that does not support or enhance ball catching skill or throwing a beanbag onto a target.

The results of 3.61 and 5.28 percent showed movement difficulties and movement at risk in Thai children, respectively. The percentage of movement difficulties is similar to that in previous studies in Columbia and Taiwan, which were 3 and 3.5 percent, respectively. However, a higher percentage was reported in studies in the UK (5–6%), Spain (9.9%) and Brazil (17.8%) [9–13]. These differences might be due to the measurement tool used, culture, and the children’s environment.

Further analysis using the Chi-square test showed significant differences between boys and girls in prevalence (movement difficulty, at risk, typical) (χ^2^ = 0.11, p = 0.001), in which Thai boys and Thai girls had different percentages. Movement difficulty and movement at risk were 1.67% and 3.61%, respectively, for boys and 1.94% and 1.67%, respectively, for girls. The prevalence of DCD in Thai children was more common in boys than girls (1.46:1). Previous studies in Korean, UK, and Spanish children found similar results between genders [9,12,28].

Further consideration of movement performance in Thai children observed the ceiling effect in dynamic balance testing (Walking-Heel-to-Toe-Forward and Hopping on Mats) and the Drawing Trail test. The administration of these items may need to be revised in order to avoid a ceiling effect and improve power in classification among age groups in Thai children.

## Limitations and suggestions

The MABC2 checklist of 30 items was not used for this study because of time constraints. Further research may translate the MABC2 checklist into the Thai Language for use by a Thai teacher. The primary purpose of this study was to us the MABC2-AB2-T to examine whether it could differentiate the movement performance from typically developed Thai children. Concurrent validity study of the MABC2-AB2 with other tests was suggested. There was a significant difference between age groups in Manual Dexterity, but no significant difference in Aiming and Catching or Balance. The Aiming and Catching and Balance subtest of the MABC2-AB2 could not differentiate from children aged 7–10 years. A further revision of the MABC2-AB2 and test administration is suggested.

## Conclusion

By using the MABC2-AB2-T to study movement performance and movement difficulties in Thai children aged 7–10 years, it was concluded that approximately 91 percent of the participants had typical movement development. Only about 9 percent of the participants showed movement difficulty (3.61%) and movement at risk (5.28%). There was a significant difference between age groups in Manual Dexterity, but none in Aiming and Catching and Balance. Ceiling effects were found in Aiming and Catching and Balance subtests. Finally, detection of movement difficulties in Thai Children with DCD is necessary, in order for them to receive appropriate early intervention.

## Implication for clinicians

The MABC2-AB2-T can differentiate Manual Dexterity from typically developed children aged between 7 and 10 years old, but cannot differentiate Aiming and Catching and Balance. Aiming and Catching and Balance seemed to improve with age increment. However, the participants showed no significant difference between age groups. It is implied that Manual Dexterity, Aiming and Catching, and Balance subtests of the MABC2-AB2-T are enough to differentiate movement performance into three categories: typical, difficult and at risk. Clinical implication from the present results benefits Thai occupational therapists in being able to examine movement performance and movement difficulty in Thai children. It is implied that the standardized test; MABC2-AB2-T, is suitable for detecting DCD among Thai children. This study is an important step towards further normative study using the MABC2-AB2-T in the Thai population.

## Abbreviation

DCD: Developmental Coordination Disorder
MABC-2: Movement Assessment Battery for Children—Second Edition
MABC2-AB2: Movement Assessment Battery for Children—Second Edition, Age Band 2
MABC2-AB2-T: Movement Assessment Battery for Children—Second Edition, Age Band 2 Thai version
UK: United Kingdom
DCDQ: Developmental Coordination Disorder Questionnaire
BOT-2: The Bruininks—Oseretsky Test of Motor Proficiency, Second Edition
